# Estimation of the incubation period and generation time of SARS-CoV-2 Alpha and Delta variants from contact tracing data

**DOI:** 10.1017/S0950268822001947

**Published:** 2022-12-16

**Authors:** Mattia Manica, Maria Litvinova, Alfredo De Bellis, Giorgio Guzzetta, Pamela Mancuso, Massimo Vicentini, Francesco Venturelli, Eufemia Bisaccia, Ana I. Bento, Piero Poletti, Valentina Marziano, Agnese Zardini, Valeria d'Andrea, Filippo Trentini, Antonino Bella, Flavia Riccardo, Patrizio Pezzotti, Marco Ajelli, Paolo Giorgi Rossi, Stefano Merler, the Reggio Emilia COVID-19 Working Group

**Affiliations:** 1 Center for Health Emergencies, Fondazione Bruno Kessler, Trento, Italy; 2 Laboratory for Computational Epidemiology and Public Health, Department of Epidemiology and Biostatistics, Indiana University School of Public Health, Bloomington, IN, USA; 3 Department of Mathematics, University of Trento, Trento, Italy; 4 Epidemiology Unit, Azienda Unità Sanitaria Locale – IRCCS di Reggio Emilia, Reggio Emilia, Italy; 5 Public Health Department, Azienda Unità Sanitaria Locale – IRCCS di Reggio Emilia, Reggio Emilia, Italy; 6 Dondena Centre for Research on Social Dynamics and Public Policy, Bocconi University, Milan, Italy; 7 Dipartimento di Malattie Infettive, Istituto Superiore di Sanità, Rome, Italy

**Keywords:** Bayesian inference, contact tracing, generation time, incubation period, SARS-CoV-2 variants

## Abstract

Quantitative information on epidemiological quantities such as the incubation period and generation time of severe acute respiratory syndrome coronavirus 2 (SARS-CoV-2) variants is scarce. We analysed a dataset collected during contact tracing activities in the province of Reggio Emilia, Italy, throughout 2021. We determined the distributions of the incubation period for the Alpha and Delta variants using information on negative polymerase chain reaction tests and the date of last exposure from 282 symptomatic cases. We estimated the distributions of the intrinsic generation time using a Bayesian inference approach applied to 9724 SARS-CoV-2 cases clustered in 3545 households where at least one secondary case was recorded. We estimated a mean incubation period of 4.9 days (95% credible intervals, CrI, 4.4–5.4) for Alpha and 4.5 days (95% CrI 4.0–5.0) for Delta. The intrinsic generation time was estimated to have a mean of 7.12 days (95% CrI 6.27–8.44) for Alpha and of 6.52 days (95% CrI 5.54–8.43) for Delta. The household serial interval was 2.43 days (95% CrI 2.29–2.58) for Alpha and 2.74 days (95% CrI 2.62–2.88) for Delta, and the estimated proportion of pre-symptomatic transmission was 48–51% for both variants. These results indicate limited differences in the incubation period and intrinsic generation time of SARS-CoV-2 variants Alpha and Delta compared to ancestral lineages.

## Introduction

The second year of the coronavirus disease-2019 (COVID-19) pandemic has been characterised by the global emergence of several lineages which were able to replace circulating ones thanks to their increased transmissibility [1]. In particular, 2021 saw the sequential rise and fall of two variants of concern, Alpha and Delta, the latter of which has been rapidly outpaced by Omicron around the end of 2021. Compared to ancestral strains, scarce quantitative information is available on several variant-specific epidemiological quantities, among which the incubation period (i.e. the time elapsed between the date of infection and symptom onset) and the generation time (i.e. the time elapsed between the date of infection of a primary case and that of a secondary case). These two quantities are especially important to define the duration of isolation for infectious individuals and of quarantines for close contacts and travellers, as well as protocols for community-based interventions such as contact tracing activities [2–4] and class/school closures [3, 5]. The knowledge of the generation time distribution also informs the estimation of the net reproduction number (i.e. the average number of new cases generated by an infectious case at a given time of the epidemic) [6], which is a key indicator for monitoring epidemic outbreaks and defining population-level measures, such as physical distancing and movement restrictions [7].

The incubation time is mostly a biologically determined parameter since it depends on virus characteristics and virus–host immunological and pathological interactions. On the other hand, the generation times that occur in a population depend on the interactions between infectious individuals and their contacts, and therefore may be subject to specific epidemiological conditions in which they are measured, including individual behaviours, environmental determinants and control measures put in place [8]. For example, observable generation times within a household are generally shorter than in the general community due to competition effects and the rapid depletion of susceptibles [8]. A distinction is therefore necessary between ‘realised’ distributions of the generation time, which are actually occurring in specific networks of contacts, and the ‘intrinsic’ distribution, i.e. the one that is expected in the general population in the absence of control interventions and local network dynamics [9]. The intrinsic generation is less sensitive to the transmissibility conditions of the epidemiological setting under study. Here, we applied a Bayesian inference approach to COVID-19 contact tracing data from the province of Reggio Emilia, Italy, during 2021 to estimate the distribution of incubation periods and generation times (both intrinsic and realised) for severe acute respiratory syndrome coronavirus 2 (SARS-CoV-2) variants Alpha and Delta.

## Methods

### Data

Contact tracing activities were carried out in the province of Reggio Emilia, Italy throughout the duration of the pandemic to mitigate the spread of SARS-CoV-2. Identified SARS-CoV-2 cases occurring in the province were confirmed via a polymerase chain reaction (PCR) assay, reported in real time to the public health service of the Reggio Emilia local health authority and isolated at home until a negative PCR test result and for a maximum of 21 days. During the study period all antigenic positive tests were confirmed with PCR. All cases were contacted via telephone to identify their close contacts. A close contact was defined as a person who stayed in the same room with a confirmed case without a face mask, or for more than 15 min at less than 2 m, between 2 days before and 10 days after symptom onset (for symptomatic cases) or diagnosis (for asymptomatic infections). Contacts were tested and quarantined at home for 10 days, if they had a negative PCR test result on that date, or for 14 days without testing [10]. All household members of a case were quarantined until a negative test after the end of the isolation period for the index case. Compliance with at least one of the tests proposed by the public health service was 97.0% during the study period (March–October 2021).

Data on test results, symptom onset date (if applicable) and setting of likely transmission were collected for all identified cases and their contacts and were linked to individual records on vaccination history (first, second and booster doses). The date of the last reported contact with any known case within a cluster (date of last exposure), as uncovered by epidemiological investigations, was also collected. Appropriate data quality checks were conducted in strict collaboration with the Reggio Emilia local health authority to minimise missing information and accurately define household clusters. A household cluster was defined as households with at least two positive individuals with a diagnosis spaced less than 25 days apart.

Since genomic information on the variant was not available, we conservatively defined two time periods where circulation of SARS-CoV-2 in the region was almost exclusively attributable (at least ~90% prevalence) to variant Alpha (1 March–30 April 2021) and to Delta (1 August–31 October 2021) [11]. Statistics of the corresponding datasets are summarised in Table 1.

**Table 1.** Descriptive statistics of SARS-CoV-2 cases in the household datasets for Alpha and Delta variants

	Alpha	Delta
Period	1 March–30 April 2021	1 August–31 October 2021
Number of cases	6272	3452
Clinical outcome (%)		
Symptomatic	3591 (57.3%)	2680 (77.6%)
Asymptomatic	2681 (42.7%)	772 (22.4%)
Gender (%)		
Male	3058 (48.8%)	1685 (48.8%)
Female	3214 (51.2%)	1767 (51.2%)
Age group (%)		
0–15 years old	1279 (20.4%)	886 (25.7%)
16–44 years old	2236 (35.7%)	1181 (34.2%)
45–64 years old	1915 (30.5%)	948 (27.5%)
65+ years old	842 (13.4%)	437 (12.6%)
Vaccination status at the end of the period (%)		
1 dose	222 (3.54%)	226 (6.5%)
2 doses	78 (1.24%)	1386 (40.2%)
3 doses	0 (0.0%)	8 (0.2%)
None	5972 (95.2%)	1832 (53.1%)
Number of households	2240	1305
Average household size (2.5–97.5 percentile range)	2.97 (2–6)	2.83 (2–5)

### Estimation of the incubation period

For the estimation of the incubation period, we selected all symptomatic cases with a date of diagnosis within either of the two periods defined for Alpha or Delta. For each case, the date of the last negative PCR test *T*_N_ and the date of last exposure *T*_L_ were used to set the limits for the earliest and last exposure, respectively. We note that the dates of test results included in the database are always referring to tests taken in response to a positivity of a contact and not tests taken autonomously by contacts for other reasons. We excluded all cases for which either date was unavailable or for which the condition *T*_N_ ≤ *T*_L_ ≤ *T*_S_, where *T*_S_ is the date of symptom onset, did not hold. The resulting sample for the estimation contains 193 observations for Alpha and 89 for Delta. We used the generalisation of the Wilcoxon–Mann–Whitney test for interval-censored data to compare the empirical data in the two samples. Two parametric distributions (gamma and Weibull) were fit to the interval-censored empirical data on the time between likely infection and the symptom onset [12, 13] using a maximum-likelihood optimisation. The best fit was selected based on the minimum Akaike information criterion [14]. Confidence intervals (CIs) for the parameters of estimated distributions were obtained from the 2.5 to 97.5 percentile range of estimates over 10 000 bootstrap samples for censored data. See Supplementary material for further details on the method and for sensitivity analyses on estimation criteria.

### Estimation of the generation time and of the serial intervals

**Fig. 1.**
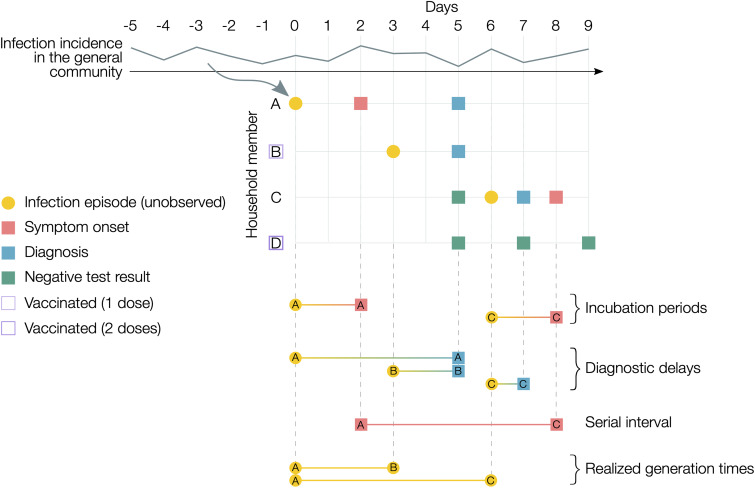
Illustrative example of actual transmission dynamics in household clusters. A household with four members, of which A was infected outside the household (in the general community) at day 0 and then transmitted to cases B (asymptomatic) and C (symptomatic), while D remained uninfected. B and D were vaccinated with 1 and 2 doses, respectively. A hypothetical epidemic curve in the general community, representing the external force of infection on household members, is reported on top of the graph. Circles indicate unobserved events; squares indicate observed events. Examples of the temporal intervals of interest for the estimates of this work are reported in the bottom part of the figure. Note that for the household serial interval and the realised household generation time, the source of infection (whether from outside the household or from a household member, and, in the latter case, which household member) is also unobserved and needs to be probabilistically reconstructed. Pre-symptomatic transmission and negative serial intervals are also possible but have not been included in this example for the sake of simplicity. The intrinsic generation time is not displayed as it represents the distribution of generation times among infections occurring in the general population in a fully susceptible population [9].

For the estimation of the generation time, we selected only household clusters for which all dates of diagnosis were included in either of the two periods defined for Alpha or Delta. To reduce the possibility of missed diagnoses in the households due to false-negative test results, we further selected households for which undiagnosed members had at least two negative test results. Figure 1 shows a schematisation of an illustrative household cluster, with the corresponding dates of infection, symptom onset, diagnosis and negative tests for individuals, as well as relevant intervals to be estimated. We adopted a Bayesian inference model for the reconstruction of transmission links in households already applied for the estimation of the generation time of the Omicron variant [15, 16]. The model exploits the temporal information on SARS-CoV-2 infections recorded in the dataset to probabilistically identify, for every case, the likely source of infection (from outside the household or from a specific household member).

We assumed the parameters for the generation time to be gamma-distributed. These parameters are simultaneously calibrated via a Markov Chain Monte Carlo approach where the likelihood of the observed data is defined mechanistically through the computation of the force of infection to which all individuals are subject over time. The force of infection takes into account the SARS-CoV-2 incidence in the general community, and the individual dates of infection and vaccination. We imputed the date of infection for each symptomatic case by subtracting from the date of symptom onset a random sample from the estimated discretised distribution of the incubation period. We then computed the diagnostic delay distribution as the distribution of delays between dates of infection and diagnosis in symptomatic individuals. Successively, we imputed the date of infection for asymptomatic cases by subtracting the date of diagnosis of a random sample from the estimated diagnostic delay distribution. For both symptomatic and asymptomatic individuals, the probability of an imputed date of infection was weighted by the probability of false-negative test results based on available test dates and results for each individual. We repeated 100 times the sampling of infection dates and re-calibrated on each resampling the Bayesian model. The 95% credible intervals (CrI) for the estimated parameters were obtained from the resulting pooled distributions. All technical details for the model and calibration are reported in the Supplementary material. Using this Bayesian approach, we could estimate at the same time both the parameters of the intrinsic generation time and the distributions of the realised household generation time and household serial interval. For each set of imputed infection dates and sample from the joint posterior distribution of parameters, we reconstructed likely transmission chains (i.e. the source of infection for each case). The distribution of realised generation times was obtained by the differences between infection dates in each inferred infector–infectee pair; correspondingly, the distribution of household serial intervals was obtained from the differences between symptom onset dates in each inferred pair of symptomatic infector–infectee.

We evaluated the robustness of model results against six sensitivity analyses (SA) encoding different assumptions in the model. In SA (a), we relaxed the assumption of the baseline model that symptomatic and asymptomatic individuals have the same distribution of diagnostic delays by considering an alternative method for inferring the date of infection of asymptomatic individuals. In SA (b) and (c), we imputed dates of infection using two alternative distributions of the incubation period previously estimated for ancestral SARS-CoV-2 lineages [13, 17]. In SA (d) we assumed a halved transmissibility for asymptomatic individuals [18]. In SA (e), we considered the possible protection from previous infection in a fraction of undiagnosed household members. Finally, in SA (f), we considered a negligible adherence of household members to quarantine (i.e. the probability of being infected outside the household was unchanged upon onset of quarantine for household members).

## Results

**Fig. 2.**
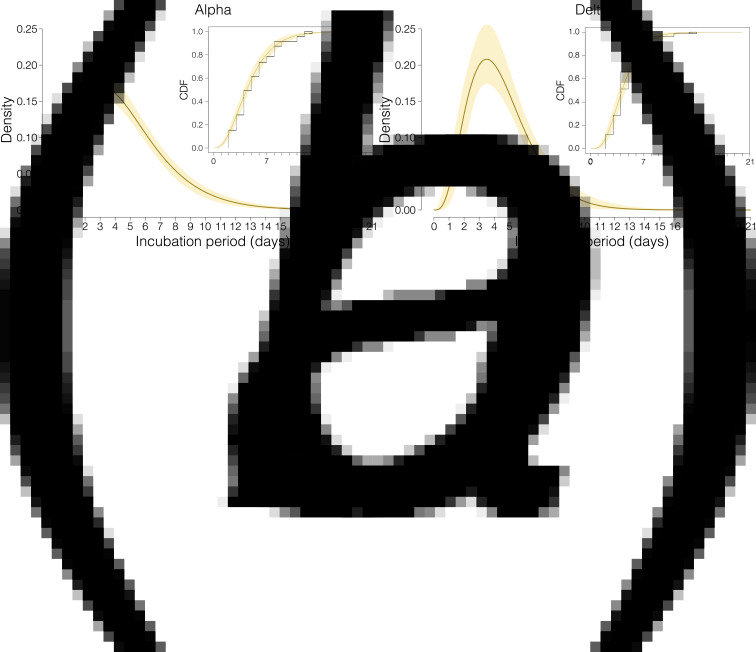
Estimation of the incubation period for the Alpha and Delta SARS-CoV-2 variants. (A) Probability density function (PDF) of the estimated distribution of incubation period for Alpha variant with 95% CI based on nonparametric bootstrap resampling of the distribution parameters (10 000 samples). Line: mean PDF; shaded area: bootstrapped pointwise 95% CI. The inset shows the cumulative distribution function (CDF) of the empirical distribution (black line) where rectangles represent areas of non-unique empirical distribution function, CDF of the distribution fitted to interval-censored data (line) and bootstrapped pointwise 95% CI on probabilities (shaded area). (B) Same as (A), but for Delta variant.

The best fit for the distributions of the incubation period was a gamma distribution, with a mean of 4.9 days for Alpha (95% CIs of the mean, CI 4.4–5.4; 2.5–97.5 percentile range of the mean distribution 1–12 days) and of 4.5 days for Delta (95% CI 4.0–5.0; 2.5–97.5 percentile range of the mean distribution 1–10 days) (Fig. 2 and Table 2). The differences between empirical distributions of incubation periods for Alpha and Delta variants were not statistically significant (Wilcoxon-type test *P* value 0.45). Unsurprisingly, the estimate for the incubation period was longer (mean: 7.3–7.4 days for Alpha and 6.2–6.3 days for Delta) and had a larger uncertainty when including in the estimation those cases for which the date of earliest exposure was unknown (Supplementary material), supporting the importance of considering only data samples for which information on the time window of exposure is more compelling.

**Table 2.** Estimates for the incubation period, diagnostic delay, intrinsic and realised generation time and household serial intervals

		Alpha	Delta
Incubation period	Mean (95% CI) [days]	4.9 (4.4–5.4)	4.5 (4.0–5.0)
	2.5–97.5 percentile range of the mean distribution [days]	1–12	1–10
	Shape mean (95% CI)	3.08 (2.56–3.86)	4.43 (3.26–6.70)
	Scale mean (95% CI)	1.58 (1.24–1.93)	1.01 (0.65–1.43)
Diagnostic delay	Mean (2.5–97.5 percentile range) [days]	7.1 (3–15)	7.1 (3–14)
Intrinsic generation time	Mean (95% CrI) [days]	7.12 (6.27–8.44)	6.52 (5.54–8.43)
	2.5–97.5 percentile range of the mean distribution [days]	1–18	1–17
	Shape mean (95% CrI)	2.53 (2.27–3.21)	2.49 (2.14–2.97)
	Scale mean (95% CrI)	2.83 (2.28–3.39)	2.63 (2.19–3.21)
Realised household generation time	Mean (95% CrI) [days]	4.41 (4.26–4.58)	4.05 (3.87–4.24)
Household serial interval	Mean (95% CrI) [days]	2.43 (2.29–2.58)	2.74 (2.62–2.88)
Pre-symptomatic transmission	Mean (95% CrI) [%]	47.8 (45.5–49.8)	50.9 (48.4–53.0)

Reported parameters of shape and scale for the incubation period and intrinsic generation time refer to a gamma distribution. The mean distribution indicates the distribution obtained using the mean value estimated for the parameters.

**Fig. 3.**
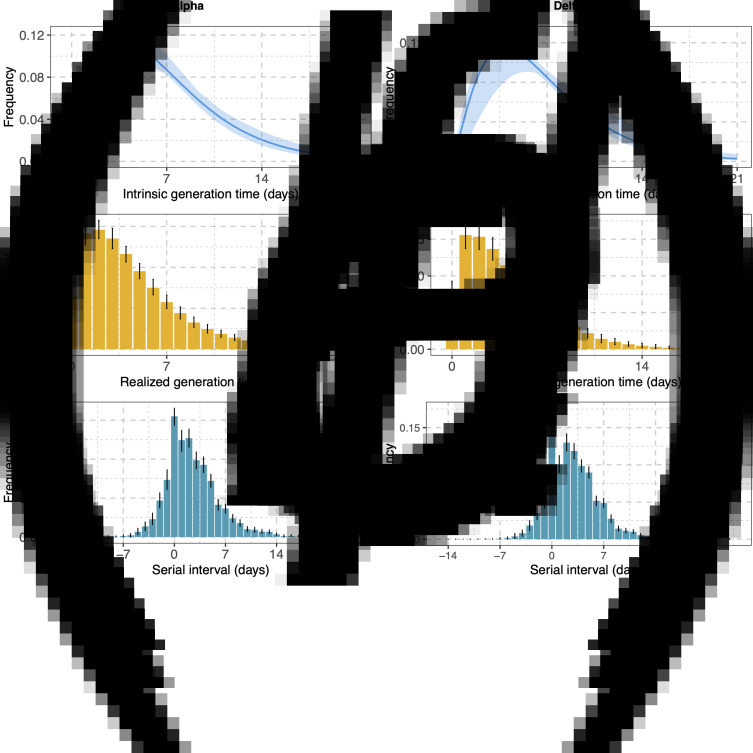
Estimates of generation times and household serial intervals for the Alpha and Delta variants. (A) Distribution of the intrinsic generation time for the Alpha variant; solid line: mean estimate; shaded area: 95% CrI; (B) same as (A), but for Delta. (C) Distribution of the realised household generation time for the Alpha variant; bars: mean estimate over all reconstructed transmission chains; vertical lines: 95% CrI across all reconstructed transmission chains; (D) same as (C), but for Delta. (E) Distribution of the household serial interval for the Alpha variant; bars: mean estimate over all reconstructed transmission chains; vertical lines: 95% CrI across all reconstructed transmission chains; (f) same as (E), but for Delta.

**Fig. 4.**
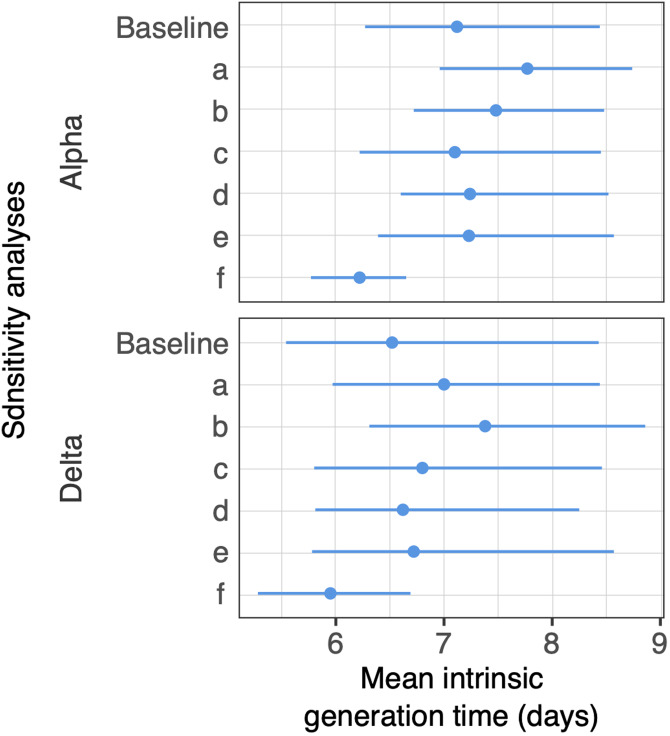
Estimates of the mean intrinsic generation time for the Alpha and Delta variants under different assumptions with respect to the baseline model: (a) uses an alternative method of imputation for the dates of infection in asymptomatic cases; (b) and (c) use different distributions of the incubation period, taken from previous estimates for ancestral SARS-CoV-2 lineages; (d) assumes a halved transmissibility for asymptomatic individuals; (e) considers the possibility of protection from previous natural infection in a fraction of undiagnosed individuals and (f) assumes no compliance of household members to quarantine. Full details on sensitivity analysis are reported in the Supplementary material.

The resulting estimated distribution of delays between infection and diagnosis (used to assign infection dates for asymptomatic individuals) had a mean of 7.14 days (2.5–97.5 percentile range: 3–15 days) for the Alpha variant (Table 2). The mean intrinsic generation time estimated for Alpha was 7.12 days (95% CrI of the mean: 6.27–8.44 days) and the mean realised household generation time was 4.41 days (95% CrI of the mean: 4.26–4.58 days) (Fig. 3 and Table 2). The mean household serial interval was 2.43 days (95% CrI of the mean: 2.29–2.58 days), with 47.8% (95% CrI 45.5–49.8) of transmission events being pre-symptomatic (i.e. secondary cases transmitted by cases who would develop symptoms after the transmission event). Sensitivity analyses yielded similar results, with the mean intrinsic generation time ranging between 6.22 and 7.77 days (Fig. 4), the mean realised household generation time ranging between 4.09 and 5.08 days and the mean household serial interval ranging between 2.14 and 2.53 days (Supplementary material).

The estimated distribution of delays between infection and diagnosis had a mean of 7.12 days for the Delta variant (2.5–97.5 percentile range: 3–14 days) (Table 2). The mean intrinsic generation time estimated for Delta was 6.52 days (95% CrI of the mean 5.54–8.43 days) and the mean realised household generation time was 4.05 days (95% CrI of the mean 3.87–4.24 days) (Fig. 3 and Table 2). The mean household serial interval was 2.74 days (95% CrI of the mean 2.62–2.88 days), with 50.9% (95% CrI 48.4–53.0) of transmission events being pre-symptomatic. Sensitivity analyses yielded similar results, with the mean intrinsic generation time ranging between 5.95 and 7.38 days (Fig. 4), the mean realised household generation time ranging between 3.84 and 4.66 days, and the mean household serial interval ranging between 2.28 and 2.76 days (Supplementary material).

## Discussion

We estimated the distribution of the incubation period and generation time for SARS-CoV-2 Alpha and Delta variants by analysing comprehensive data collected during contact tracing activities in the province of Reggio Emilia, Italy, throughout 2021. We found no statistical difference for the duration of the incubation period for Alpha (mean: 4.9 days) and Delta (mean: 4.5 days) variants. Both estimates are very close to those reported for the same variants in a recent extensive meta-analysis (Alpha: 5.0 days; Delta: 4.4 days) [19] and in line (albeit slightly shorter) with those obtained for the ancestral lineages [12, 17, 20, 21]. We did not evaluate the dependence between age and incubation periods for Alpha and Delta variants, which was previously evaluated for ancestral lineages [22].

Our estimates of the mean generation time for both Alpha (mean: 7.12 days) and Delta (mean: 6.52 days) are compatible with previous estimates for ancestral lineages [23–27], including a previous estimate for Italy of 6.68 days [28]. We also found comparable household serial intervals between Alpha (mean: 2.43 days) and Delta (mean: 2.74 days) with similar proportions of pre-symptomatic transmissions (about 50% for both variants). Previous estimates on both ancestral lineages and Alpha and Delta variants are highly variable (due to the high sensitivity of these parameters to epidemiological conditions of the study settings) and ranged between 1.8 and 7.5 days [12, 13, 17, 29, 30] for the serial interval and between 13% and 65% [12, 13, 21] for the proportion of pre-symptomatic transmission.

Estimates of the intrinsic generation time may depend on epidemiological specificities of the geographical setting from which the data are collected, as well as by the inference method. For instance, a study conducted in England estimated, using a different approach, a shorter mean intrinsic generation time for the Alpha (5.5 days) and Delta variants (4.7 days) [30]. Given its potential sensitivity to local factors, we point out the need to obtain country-specific estimates of the distribution of the generation time. For what concerns Italy, this study suggests the adequacy of epidemiological analyses (i.e. computation of reproduction numbers; modelling estimates) performed by assuming a distribution of the generation time similar to ancestral lineages.

A main strength of this work consists in the very large population-based dataset that comprehensively covers household clusters observed in the province of Reggio Emilia. The protocol for tracing and testing contacts was the same in the Alpha and Delta periods. Thanks to efforts by public health officials, a high compliance to testing was achieved, with only 3% of individuals refusing to be tested; in addition, all household members of cases were tested on the same date of the first diagnosis in the household. To minimise the possibility that our data contain clusters due to other variants, we selected two periods where Alpha and Delta were largely dominant [12]. However, for the Alpha period a residual circulation (7–8% prevalence) of the Gamma variant was detected in the Emilia-Romagna region [11, 31]. The estimates of the intrinsic generation time can be compared across periods with different vaccination coverage since the model includes susceptibility and transmissibility variations according to the individual's vaccination history. A limitation in the estimation of the incubation period was the use of censored interval data under the assumption that the date of infection was bounded by the date of last negative test and the date of last exposure, as both dates may suffer potential biases. In some cases, the date of last negative test may be a too stringent limit for the date of first exposure, as a test can provide false-negative results if performed in the days immediately successive to the date of infection. On the other hand, the date of last exposure may be incorrectly recorded if a case broke from isolation/quarantine and did not report further contacts to the tracing team for fear of administrative fines. These biases may have an impact on the estimation of incubation periods. However, the closeness of our estimates to results of a recent extensive meta-analysis [19] and the high level of collaboration and trust between the population and the contact tracing teams within this study (as witnessed by the high level of compliance to offered tests) suggest that such biases may be mild in our data.

A limitation of the model for the estimation of the generation time is its reliance on assumptions for the dates of infection of infected individuals. Ideally, these could be inferred as nuisance parameters in the model, but this is computationally unfeasible with the large number of cases within this study. Therefore, dates of infection were imputed multiple times based on the distribution of the incubation period [32]. The same intrinsic limitation of the unobservability of infection times is shared by all transmission chain reconstruction models, but there are now several examples where these models have been proven to correctly identify the transmission dynamics of infectious outbreaks [32–36]. Estimates were substantially robust with respect to different methods of imputation and different distributions of the incubation period (Fig. 4 and Supplementary material). Thus, potential biases in the estimate of the incubation period reported above are not expected to propagate to the generation time. A specific limitation of this study was the lack of information about previous SARS-CoV-2 infection in undiagnosed individuals. In the main analysis we assumed that all undiagnosed individuals did not have a pre-existing protection from natural immunity. However, in a sensitivity analysis, we show that assuming full protection from previous infection in a fraction of undiagnosed individuals hardly affects our results (Supplementary material). Another specific limitation is that we assumed 100% compliance to quarantine protocols (i.e. that household members quarantined after diagnosis of another member could only be infected within the household). A sensitivity analysis where quarantines of household members are not considered (i.e. 0% compliance) yielded similar results to the ones illustrated in the main analysis (Supplementary material).

## Conclusion

Results from this study suggest that the length of the incubation period and generation time for Alpha and Delta variants were comparable to those of the ancestral lineages. These findings provide support to the recommendations of adopting duration of quarantine, isolation and contact tracing operations similar to those for the ancestral lineage. This work also confirms the suitability of the adopted method for estimating the incubation periods and generation times on further emerging variants of concern, provided that high-quality contact tracing data are available.

## Data Availability

The data that support the findings of this study are openly available on the online repository figshare, doi:10.6084/m9.figshare.19802659.

## Supplementary material

For supplementary material accompanying this paper visit https://doi.org/10.1017/S0950268822001947. click here to view supplementary material
