# Supplementary material for: Estimation of the incubation period and generation time of SARS-CoV-2 Alpha and Delta variants from contact tracing data
Source: Epidemiol Infect. 2022 Dec 16;151:e5. doi: 10.1017/S0950268822001947 (PMC9837419; doi:10.1017/S0950268822001947)
Supplement: Supplementary file 1 [file S0950268822001947sup001.pdf]

Supplementary Material

Estimation of the incubation period and generation time of SARS-CoV-2 Alpha and Delta variants from contact tracing data

Mattia Manica [a,*], Maria Litvinova [b,*], Alfredo De Bellis [a,c], Giorgio Guzzetta [a], Pamela Mancuso [d], Massimo Vicentini [d], Francesco Venturelli [e], Eufemia Bisaccia [e], Ana I. Bento [b], Piero Poletti [a], Valentina Marziano [a], Agnese Zardini [a], Valeria d'Andrea [a], Filippo Trentini [a,f], Antonino Bella [g], Flavia Riccardo [g], Patrizio Pezzotti [g], Marco Ajelli [b,#], Paolo Giorgi Rossi [d,#], Stefano Merler [a,#,%] and the Reggio Emilia COVID-19 Working Group.

a. Center for Health Emergencies, Fondazione Bruno Kessler, Trento, Italy
b. Laboratory for Computational Epidemiology and Public Health, Department of Epidemiology and Biostatistics, Indiana University School of Public Health, Bloomington, IN, USA
c. Department of Mathematics, University of Trento, Trento, Italy
d. Epidemiology Unit, Azienda Unità Sanitaria Locale – IRCCS di Reggio Emilia, Reggio Emilia, Italy
e. Public Health Department, Azienda Unità Sanitaria Locale – IRCCS di Reggio Emilia, Reggio Emilia, Italy
f. Dondena Centre for Research on Social Dynamics and Public Policy, Bocconi University, Milan, Italy
g. Dipartimento di Malattie Infettive, Istituto Superiore di Sanità, Rome, Italy

[*] joint first authors
[#] joint senior authors
[%] corresponding author: merler@fbk.eu

The following are members of the Reggio Emilia Covid-19 Working Group: Emanuela Bedeschi, Cinzia Perilli, Nadia Montanari, Francesca Pia Leonetti, Nicoletta Patrignani, Letizia Bartolini, Francesca Roncaglia, Isabella Bisceglia, Valeria Cenacchi, Barbara Braghiroli, Anna Pezzarossi.

Supplementary Material

Table of Contents

1. Estimation of the incubation period 3

Sensitivity analyses 4

2. Imputation of dates of infection 6

3. Estimation of the generation time distribution and inference of transmission links 7

4. Additional results of the baseline model 10

Statistics on reconstructed transmission links 10

Stability of the attributed source of infection 11

5. Sensitivity Analyses 11

a) Imputation of dates of infection in asymptomatic cases 12

b) Distribution of the incubation period – I 13

c) Distribution of the incubation period – II 14

d) Reduced transmissibility for asymptomatic individuals 16

e) Protection from previous infection in a fraction of undiagnosed household members 17

f) No protection from infection outside the household during quarantine 18

References 20

1. Estimation of the incubation period

For the estimation of the incubation period, we considered observations on individuals who were contacts of an index case and who later became symptomatic and diagnosed with SARS-CoV-2. Among these, we selected cases having a diagnosis in either of the selected study period for Alpha (1021 cases) or Delta (519 cases). The date of symptom onset and the date of last exposure was available for all symptomatic cases. For each case, the potential incubation period was bounded by the date of the latest negative test result before the diagnosis (earliest possible exposure) and by the date of the last exposure. By considering the latest negative test as a proxy for the earliest possible exposure, we assume the test to have a perfect sensitivity, i.e. we neglect possible negative false results which are especially probable in the earliest days after infection. We excluded 172 Alpha cases and 53 Delta cases for which the information on exposure were conflicting (e.g., last negative test successive to the last reported exposure), 298 Alpha cases and 173 Delta cases for which the date of last exposure was successive to symptom onset, and 358 Alpha cases and 204 Delta cases for which only a date of last exposure was available, obtaining 193 Alpha cases and 89 Delta cases for the main analysis (see Figure S1 for the sample selection). The resulting censored intervals of the possible incubation periods are reported for all cases in Figure S2.

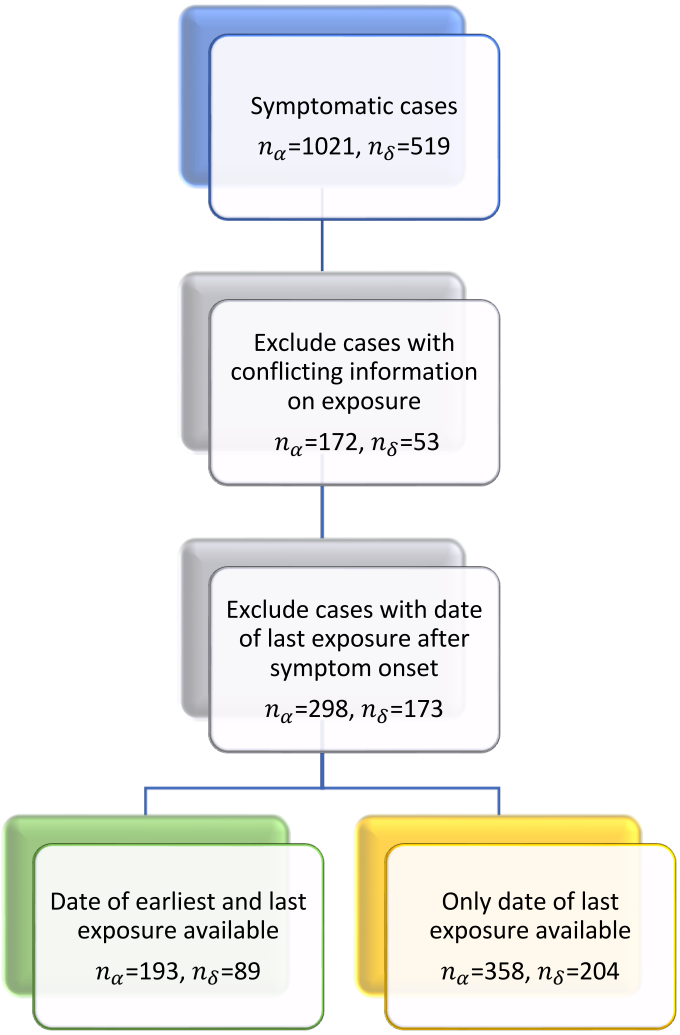

Figure S1. Workflow of sample selection. Gray boxes represent exclusion steps. The green box shows the sample used for the main analysis. The yellow box shows the additional sample used for a sensitivity analysis. n_α represents the sample size for Alpha variant, n_δ represents the sample size for Delta variant

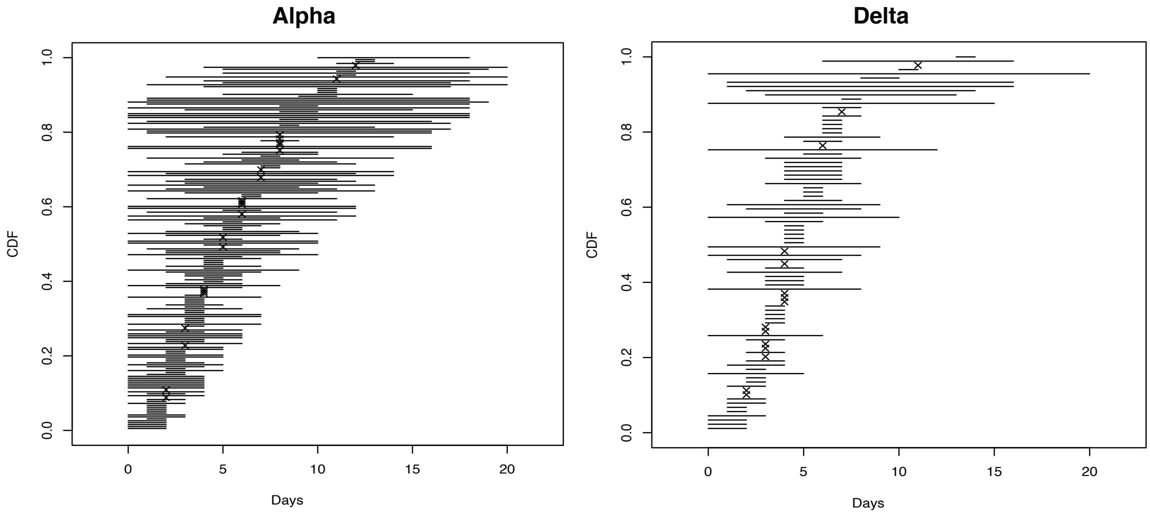

Figure S2. Incubation period censored data. Interval censored and non-censored observations for each case are ordered by their mid-points.

We estimated both Gamma and Weibull distributions using the censored data. Maximum likelihood estimations of the distribution parameters were calculated by using the *fitdistrplus* package in R. Direct optimization of the log-likelihood is performed using general-purpose optimization based on Nelder–Mead, quasi-Newton algorithm for both Gamma and Weibull distributions. Nonparametric bootstrap resampling was used to simulate uncertainty in the parameters of the estimated distributions. Results of the estimation procedure described in the main text are presented in Table S1.

Table S1. Estimated distribution of the incubation period. SD: Standard Deviation. AIC: Akaike Information Criterion

Variant	Distribution	Parameters: mean (SD)	Mean distribution (days)			AIC score
			Mean	SD	2.5 to 97.5 percentile range	
Alpha (N=193)	Gamma	shape = 3.08 (0.39), rate = 0.63 (0.084)	4.9	2.8	1.0 – 11.7	506.9
	Weibull	scale = 5.52 (0.27), shape = 1.83 (0.13)	4.9	2.8	0.7 – 11.3	510.7
Delta (N=89)	Gamma	shape = 4.43 (0.76), rate = 0.99 (0.18)	4.5	2.1	1.3 - 9.6	261.3
	Weibull	scale = 5.09 (0.30), shape = 2.10 (0.18)	4.5	2.2	0.9 - 9.5	267.1

Sensitivity analyses

As a first sensitivity analysis, we added the observations with only the date of last exposure being available (see Figure S1). For cases with unknown date of earliest possible exposure, we set the maximum boundary of the incubation period to 21 days before the symptom onset. Figure S3 shows the censored data used in this estimation. This increased both the sample size and the uncertainty regarding the earliest possible exposure, naturally increasing the average of the estimated incubation period (Table S2). This shows the importance of considering only data samples for which information on the time window of exposure is more compelling. Then, we repeated the main analysis and the sensitivity analysis above after selecting cases falling within the Alpha or Delta period based on the date of symptom onset rather than on the date of diagnosis, obtaining similar results to the corresponding analyses above.

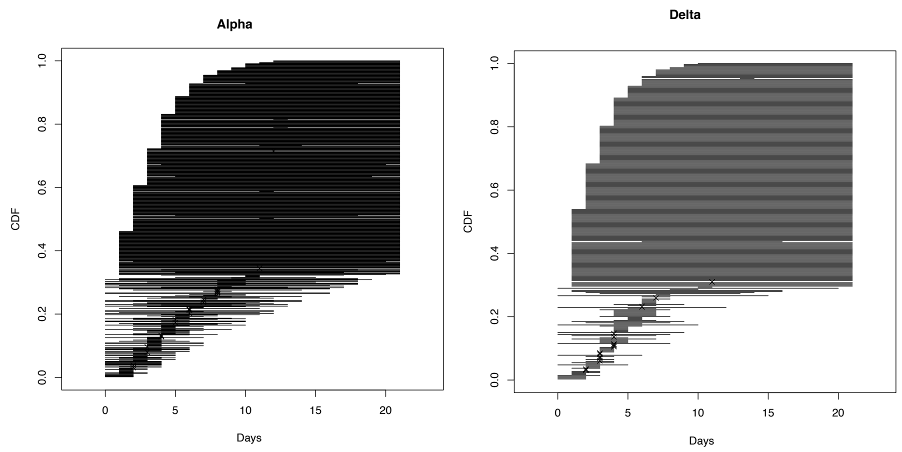

Figure S3. Incubation period censored data, including observations with no information on earliest possible exposure (sensitivity analysis A). Interval censored and non-censored observations for each case are ordered by their mid-points.

Table S2. Estimated distribution of incubation period in sensitivity analyses. SD: Standard Deviation. AIC: Akaike Information Criterion

Variant	Distribution	Parameters: mean (SD)	Mean distribution (days)			AIC score
			Mean	SD	2.5 to 97.5 percentile range	
A) Date of the last exposure available (independently from availability of earliest exposure)						
Alpha (N=551)	Gamma	shape = 3.75 (0.31), rate = 0.51 (0.05)	7.3	3.8	1.9 - 16.6	770.9
	Weibull	scale = 8.33 (0.29), shape = 2.23 (0.12)	7.4	3.5	1.6 - 15.0	764.7
Delta (N=293)	Gamma	shape = 4.58 (0.60), rate = 0.73 (0.12)	6.3	2.9	2.0 - 13.2	376
	Weibull	scale = 7.07 (0.33), shape = 2.49 (0.19)	6.3	2.7	1.7 - 12.0	380.2
B) As main analysis, but cases are assigned to variant via date of symptom onset						
Alpha (N=187)	Gamma	shape = 3.12 (0.40), rate = 0.65 (0.087)	4.8	2.7	1.0 - 11.4	495.5
	Weibull	scale = 5.45 (0.27), shape = 1.84 (0.13)	4.8	2.7	0.7 - 11.1	499.2
Delta (N=89)	Gamma	shape = 4.70 (0.81), rate = 1.04 (0.19)	4.5	2.1	1.4 - 9.4	255.9
	Weibull	scale = 5.10 (0.30), shape = 2.14 (0.19)	4.5	2.2	0.9 - 9.3	263
C) As sensitivity analysis A), but cases are assigned to variant via date of symptom onset						
Alpha (N=546)	Gamma	shape = 3.71 (0.31), rate = 0.50 (0.049)	7.4	3.8	1.9 - 16.5	769
	Weibull	scale = 8.45 (0.29), shape = 2.21 (0.12)	7.5	3.6	1.5 - 15.4	763.1
Delta (N=286)	Gamma	shape = 4.75 (0.64), rate = 0.76 (0.12)	6.2	2.9	1.9 - 12.8	367.7
	Weibull	scale = 7.04 (0.32), shape = 2.51 (0.19)	6.3	2.7	1.6 - 12.0	373.3

2. Imputation of dates of infection

The task of reconstructing transmission chains must overcome the intrinsic limitation of the unobservability of transmission chains. We use available evidence to probabilistically impute plausible infection dates for all SARS-CoV-2 cases in our dataset. We combine observed dates of symptom onset, diagnosis, and negative test results with available knowledge on incubation periods and the probability of testing positive over time for infected individuals.

First, we impute the dates of infection for all symptomatic cases.

Let T_D be the date of diagnosis (when the individual tested positive), $T_{N,n}$ the date of the n-th negative test before diagnosis, and T_S the date of symptom onset; we define the following probability P_I of being infected on day T_I to be proportional to the product of three probabilities:

- the probability of having an incubation period equal to T_s-T_I days;
- the probability of testing positive at the date of diagnosis given infection at day T_I;
- the probability of testing negative (including false negatives) at all the dates of negative tests given infection at day T_I;

This can be summarized by the following equation:

$$P_I(T_I) = f(T_D - T_I) \cdot \prod_n [1 - f(T_{N,n} - T_I)] \cdot P_S(T_s - T_I) \qquad \text{(Eq. 1)}$$

Where $f(t)$ is the probability of a SARS-CoV-2 case of testing positive after a time t since infection and $P_S(t) = \int_t^{t+1\,day} p_S(\tau)d\tau$ is the discretized version of the probability density function of the incubation period $p_S(t)$. For $p_S(t)$ we use the average variant-specific estimate from contact tracing data in Reggio Emilia defined by the algorithm above as a baseline, and two previous alternative estimates on ancestral lineages [S2, S3] as sensitivity analyses (see Section S5-b and S5-c). For $f(t)$, we use a previously estimated piecewise logistic function with one breakpoint [S1], also discretized at intervals of one day. For each symptomatic case, a time of infection T_I is sampled from $P_I(t)$; note that this sampling allows for possible false negative results in dates $T_{N,n}$. The sample is repeated K = 100 times.

For asymptomatic cases, we cannot use the information on the incubation period given that no date of symptom onset is defined. Therefore, we use the imputed dates of infection for symptomatic cases to define a distribution of diagnostic delays $P_D(x)$, defining the probability of being diagnosed after x days from infection. An empirical approximation of $P_D(x)$ will be given, for any x, by the fraction of all instances across the K stochastic samples for which the diagnostic delay $T_R = T_D - T_I$ is equal to x. A gamma function p_D for the probability density function of the diagnostic delay is then fitted to the empirical distribution using a maximum likelihood approach and then discretized as above to obtain $P_D(x) = \int_x^{x+1\,day} p_D(\tau)d\tau$. The infection date of asymptomatic cases can then be sampled from the following probability

$$P_I(T_I) = f(T_D - T_I) \cdot \prod_n [1 - f(T_{N,n} - T_I)] \cdot P_D(T_D - T_I) \qquad \text{(Eq. 2)}$$

Equation 2 has the same rationale as that of equation one, except that instead of the incubation period term we consider the probability of having a diagnostic delay equal to T_D-T_I, assuming that the distribution of diagnostic delays for asymptomatic cases is the same as for symptomatic cases. Because this assumption cannot be tested, we use as a sensitivity analysis an alternative method where only the probabilities of negative and positive tests are used to define the P_I (see Section S5-a). The sampling of infection times is repeated K times also for asymptomatic cases.

Assuming the imputation of incubation periods is correct, we obtain that 12.6% (95%Credible Intervals, CrI: 11.6-13.7%) of negative tests is a false negative result for the Alpha variant and 18.1% (95%CrI: 16.5-19.5%) for the Delta variant. This result was used to define the criterium of inclusion for households where undiagnosed cases have at least two negative tests, in order to reduce the fraction of undiagnosed positive cases to negligible levels (1.6% for Alpha and 3.3% for Delta) for the purpose of this analysis.

Figure S4 reports the estimated empirical and fitted distributions of diagnostic delays for variants Alpha and Delta.

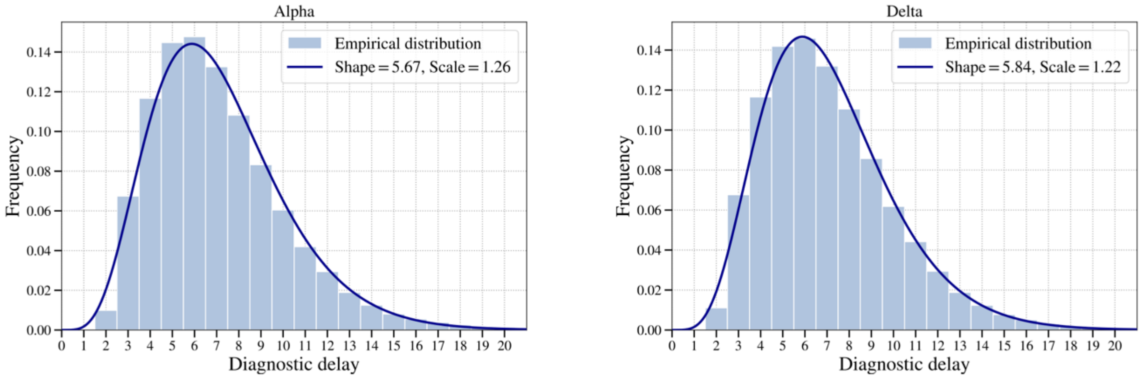

Figure S4. Empirical and fitted distribution of the diagnostic delay P_D, estimated from symptomatic cases. Left: Alpha variant; Right: Delta variant. The histograms represent the empirical distribution given the imputed infection times for symptomatic individuals. The curves represent the fit of gamma functions.

3. Estimation of the generation time distribution and inference of transmission links

The model adopted in this work extends the approach previously proposed in [S4]. We assumed that, at any time t, a susceptible individual j within a household is exposed to a force of infection composed of two components:

$$\lambda_j(t) = \lambda_j^o(t) + \lambda_j^h(t) \quad \text{(Eq. 3)}$$

Where $\lambda_j^o(t)$ represents the force of infection from the general community outside the household, and $\lambda_j^h(t)$ represents the one from infected members inside the household; this considers the possibility that an individual can be infected either within the household by one of its members or in the general community.

We now specify the two components. First, we describe the force of infection at time t from the general community $\lambda_j^o(t)$ as the forces of infection exerted at day t on the individual j by all potential infectors in the general community. We define the force of infection from the general community as given by the sum of the individual forces of infection from all cases that were infected at any day z before t. The force of infection from each candidate infector was proportional to the relative susceptibility of j according to his vaccination status and to the probability of infecting t-z days after infection; we also considered the possibility that j was in quarantine/isolation at home at day t and therefore could not have contacts with the general community. Therefore, we define $\lambda_j^O(t)$ as follows:

$$\lambda_j^O(t) = \sum_{z \in 0..t} \psi \, Inc(z) \, \chi_j(t) \Gamma(t-z; a, b) q_j(t) \quad \text{(Eq. 4)}$$

Where:
- ψ is an unknown transmission rate from the general community;
- $Inc(z)$ is the number of newly infected cases at time z outside the household of j
- $\chi_j(t)$ represents the relative susceptibility of individual j and changes over time t depending on the dates of vaccination of j;
- $\Gamma(t; a, b)$ represents the distribution of the intrinsic generation time at day t after infection, for which we assumed a discretized Gamma distribution with scale a and shape b; in particular, given $g(t; a, b)$ the continuous Gamma probability distribution, $\Gamma(t; a, b) = \int_t^{t+1} g(\tau; a, b) d\tau$.
- the term $q_j(t)$ is an on/off function that is 0 when the household of j is in quarantine and 1 otherwise. For each household, a quarantine of 14 days is started after the first diagnosis and reinstated for a further 14 days every time there is a new diagnosis after the previous quarantine has ended.

Since $Inc(z)$ is unknown due to underreporting, we considered the epidemic curve by date of symptom onset for the province of Reggio Emilia in the Italian integrated surveillance system [S5, S6], $I(z)$, which is proportional to $Inc(z)$ via an unknown reporting parameter u: $I(z) = u\ Inc(z)$.
Thus, equation 4 becomes:

$$\lambda_j^o(t) = \sum_{z\ \in\ 0..t} \frac{\psi}{u}\ I(z)\ \chi_j(t)\Gamma(t-z;a,b)q_j(t) \qquad \text{(Eq. 4b)}$$

Because ψ and u cannot be estimated at the same time due to their collinearity, we estimate a single free parameter α that is a scaling factor accounting for both underreporting of cases and the transmissibility from the general community. This allows to make the problem tractable at the cost of losing the interpretability on the estimated value of α.

Similarly, we describe the force of infection at time *t* from the general community $\lambda_j^h(t)$ as the sum of all forces of infection exerted on the individual j by each household members with an earlier date of infection. The force of infection $\lambda_{j,i}^h(t)$ from one household member *i* was proportional to the relative transmissibility of *i* at time t (according to the vaccination status of *i*), the relative susceptibility of j at time t (according to the vaccination status of *j*), and to the probability of transmitting t-$T_{I,i}$ after the date of infection $T_{I,i}$. Therefore, we define $\lambda_j^h(t)$ as:

$$\lambda_j^h(t) = \sum_{i \in H_j} \lambda_{j,i}^h(t) = \sum_{i\ \in\ H_j} \beta\rho_i(t)\chi_j(t)\Gamma\big(t-T_{I,i};a,b\big) \qquad \text{(Eq. 5)}$$

where:

- *i* is an index running over the set H_j of infected household members of individual *j*;
- $\rho_i(t)$ represents the relative transmissibility of individual *i*, and changes over time *t* depending on the dates of vaccination of *i*;
- β is a free parameter scaling the transmissibility inside households.

For the relative susceptibility, we assumed that each dose may reduce the susceptibility to a given value 14 days after inoculation; protection of each dose starts to wane immediately, following an exponential function, increasing again the susceptibility over time. When the booster dose (third dose) is administered, we assume no waning (note that the booster dose started to be administered in Italy towards the end of the Delta study period, with only 3 individuals in our data having received it):

$$\chi_j(t) = \begin{cases} 1 & if\ t < t_{v,1}+14 \\ 1-\eta^{(1)}\ e^{-w(t-t_{v,1}-14)} & if\ t_{v,1}+14 \le t < t_{v,2}+14 \\ 1-\eta^{(2)}e^{-w(t-t_{v,2}-14)} & if\ t_{v,2}+14 \le t < t_{v,3}+14 \\ 1-\eta^{(3)} & if\ t \ge t_{v,3}+14 \end{cases} \qquad \text{(Eq. 6)}$$

Where $t_{v,d}$ is the date of vaccination dose *d*, $\eta^{(d)}$ are the initial effectiveness of dose *d* (i.e., 14 days after vaccination) against the considered variant, and w is the waning rate of vaccine protection. Estimates of vaccine effectiveness and waning rate were obtained from a large-scale retrospective cohort study on the Italian population [S7, S8] and reported in Table S3.

Table S3. Parameters for vaccine effectiveness and waning.

Parameter	Unit	Alpha	Delta
**Initial effectiveness of dose 1 $\eta^{(1)}$**	%	49.2	49.4
**Initial effectiveness of dose 2 $\eta^{(2)}$**	%	81.9	80.2
**Effectiveness of the booster dose $\eta^{(3)}$**	%	-	80.2
**Waning rate** *w*	$days^{-1}$	0	1/227

For the relative transmissibility, we assumed a reduction by ρ = 50% after 14 days from the first dose [S9, S10]:

$$\rho_i(t) = \begin{cases} 1 & if\ t < t_{v,1} + 14 \\ \rho & if\ t \geq t_{v,1} + 14 \end{cases} \quad \text{(Eq. 7)}$$

The model assigns a source of infection k_j for all cases by choosing from either a generic source outside the household or from an infectious household member in H_j, with probability proportional to the contribution of each source to the total force of infection $\lambda_j(T_{I,j})$ at the time $T_{I,j}$ at which j was infected.
The probability for an individual j of being infected at time $T_{I,j}$, L_j, is given by the product of the probability of being infected by the assigned source of infection on that day (P_j) and the probability of not being infected up until that day (Q_j). The overall likelihood of the observations given parameter set $\theta = (\alpha, \beta, a, b)$ and the assigned sources of infection k_j is given by the product of individual probabilities L_j:

$$L(\theta, k_j) = \prod_j L_j = \prod_j P_j Q_j \quad \text{(Eq. 8)}$$

where

$$P_j = \begin{cases} \lambda_j^o(\mathrm{T_{I,j}}) & \text{if } k_j \text{ is outside the household} \\ \lambda_{j.i}^h(\mathrm{T_{I,j}}) & \text{if } k_j \text{ is household member } i \\ 1 & \text{if } j \text{ is uninfected} \end{cases} \quad \text{(Eq. 9)}$$

For infected individuals, Q_j is the probability that *j* has not been infected until $t_{I,j}$, namely $Q_j = e^{-\int_0^{t_{I,j}} \lambda_j(t)dt}$. For uninfected individuals, it is the probability that *j* has never been infected, $Q_j = e^{-\int_0^{\infty} \lambda_j(t)dt}$.

We estimated the unknown parameters θ and the source of infection k_j for all cases using a Monte Carlo Markov Chain (MCMC) procedure. We considered uniform prior distribution for all parameters (α: Uniform(10^{-8}, 10^{-4}); β: Uniform(0.1, 4); a: Uniform(0.1, 5); b: Uniform(0.1, 5). At each step, all parameters in θ are updated using reversible normal jumps. Z=500 samples from the posterior distributions obtained by the MCMC for each of the K=100 samples were pooled together to obtain the final parameter distribution and the distribution of the sources of infection for each case. Each sample of the joint distribution of the sources of infection constitutes a possible reconstructed transmission chain.

4. Additional results of the baseline model

Table S4 shows statistics on the posterior distributions of parameters for the intrinsic generation time.

Table S4. Statistics on the posterior distributions of parameters for the intrinsic generation time in the baseline model.

Alpha	Shape variance	0.04
	Scale variance	0.08
	Covariance	-0.032
Delta	Shape variance	0.05
	Scale variance	0.07
	Covariance	-0.024

Statistics on reconstructed transmission links

Given the set of 50,000 reconstructed transmission chains, it is possible to compute descriptive statistics on the number of infections acquired within or outside the household accounting for household size (Table S5). We obtained that the average per-household number of infections contracted from the general community was 1.18 (95%CrI 1.16 – 1.22) during the Alpha period and 1.11 (95%CrI 1.08 – 1.14) during the Delta period. The average number of secondary infections generated by a positive case was 0.64 (0.63 – 0.65) during the Alpha period and 0.60 (0.59 – 0.61) during the Delta period. Table S5 shows how the model reconstructed transmission links within households with different numbers of cases.

Table S5. Statistics for the model-based reconstruction of transmission links in households by number of SARS-CoV-2 cases. Reported numbers are the average and their 95% CrI, in bold the total number of households in the sample.

	Alpha		Delta	
	Number	%	*Number*	%
**Households with 2 SARS-CoV-2 cases**	***1158***	**100**	***748***	**100**
- Both infected in the general community	*94 (75-121)*	8 (6-10)	*48 (35-63)*	6 (5-8)
- One infected the other	*1064 (1034-1083)*	92 (89-94)	*700 (685-713)*	94 (92-95)
**Households with 3 SARS-Cov-2 cases**	***611***	**100**	***338***	**100**
- All infected in the general community	*2 (0-6)*	0 (0-1)	*1 (0-3)*	0 (0-1)
- One transmission, 2 infected in the general community	*75 (58-94)*	12 (9-15)	*31 (20-42)*	9 (6-12)
- Two transmissions, same infector (1 generation)	*248 (221-277)*	41 (36-45)	*143 (121-167)*	42 (36-49)
- Two transmissions, different infectors (2 generations)	*286 (251-317)*	47 (41-52)	*163 (139-188)*	48 (41-56)
**Households with 4 or more SARS-Cov-2 cases**	***471***	**100**	***219***	**100**
- All infected in the general community	*0 (0-1)*	0 (0-0)	*0 (0-0)*	0 (0-0)
- One transmission	*58 (42-77)*	12 (9-16)	*29 (19-41)*	13 (9-19)
- Two transmissions	*241 (221-261)*	51 (47-55)	*117 (104-129)*	53 (47-59)
- Three or more transmissions	*171 (136-207)*	36 (7-44)	*73 (52-94)*	33 (24-43)

Stability of the attributed source of infection

For each case, we considered the distribution of the sources of infection attributed by the model through the reconstructed chains of transmission and evaluated its stability. We categorized cases according to whether its source of infection was consistently (i.e., more than 75% of the times over the Z sampling of infector and K sampling of infectious dates) attributed to:

- the same household member;
- transmission within household but from different potential infectors;
- transmission in the general community.

The setting of transmission was uncertain (less than 75% consistency in attribution) in about 40% of cases (Figure S5) in both the Alpha and Delta periods. This generally happened when two or more cases in a household had close diagnosis dates, so that either could have been infected in the general community and then transmitted to the other, or both could have been infected in the general community, depending on the assigned dates of infection.

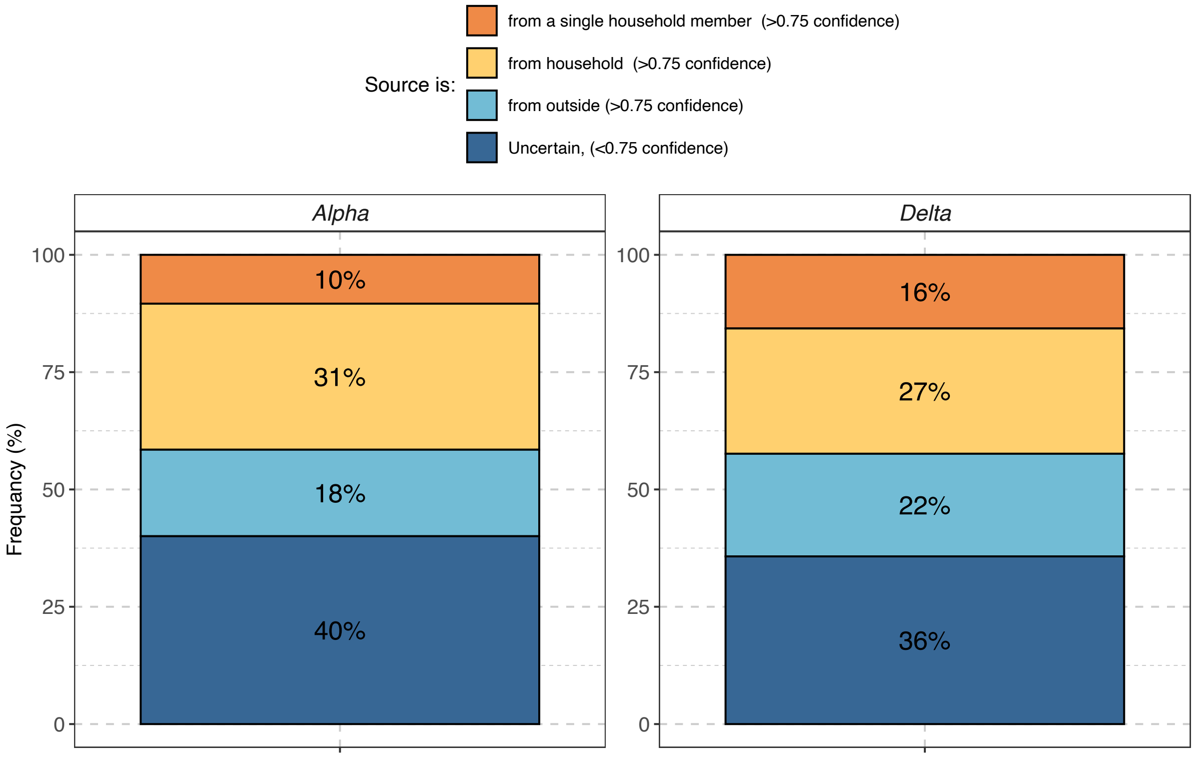

Figure S5. Consistency in the attribution of the infector or the infector setting. The stacked barchart represents the proportion of individuals that were consistently (more than 75% of the times across Z sampling of sources and K sampling of infectious dates) or inconsistently attributed to either category.

5. Sensitivity Analyses

We performed six sensitivity analyses (SA) to test the robustness of model results against different model assumptions. The first three SA (*a-c*) impact on the main unknown of the data, i.e. the imputed infectious periods of cases; the fourth (*d*) considers a reduced transmissibility for asymptomatic individuals; the fifth (*e*) evaluates the possibility that a fraction of undiagnosed individuals were fully protected from infection from previous natural immunity: the sixth (*f*) assumes that any effort to quarantine positive cases would not impact the force of infection from outside the household (*ie* $q(t) = 1$ for any value of t in equation 4b)

a) Imputation of dates of infection in asymptomatic cases

In the baseline method for the imputation of dates of infection, we implicitly assumed that symptomatic and asymptomatic cases have the same diagnostic delay distribution. We assess the impact of this assumption by considering an alternative method where the date of infection of asymptomatic individuals was assigned only on the basis of information on diagnostic date and negative test results. In this additional procedure the factor depending on P_D is removed from Equation 2, resulting in:

$$P(j) = f(t_D - j) \cdot \prod_n [1 - f(n - j)] \qquad \text{(Eq. 10)}$$

Table S6 and Figure S6 show that results obtained in this sensitivity analysis are in line with the baseline.

Table S6. Estimates for the intrinsic and realized generation time and serial intervals using an alternative method for the imputation of infection dates for asymptomatic individuals.

		ALPHA	DELTA
INTRINSIC GENERATION TIME	mean (95%CrI) [days]	7.77 (6.96-8.74)	7 (5.97-8.44)
	shape mean (95%CrI)	2.43 (2.12-3.15)	2.33 (2.03-2.78)
	scale mean (95%CrI)	3.22 (2.62-3.65)	3.02 (2.4-3.69)
REALIZED GENERATION TIME	mean (95%CrI) [days]	5.08 (4.87-5.33)	4.39 (4.22-4.59)
SERIAL INTERVAL	mean (95%CrI) [days]	2.53 (2.37-2.72)	2.76 (2.64-2.88)

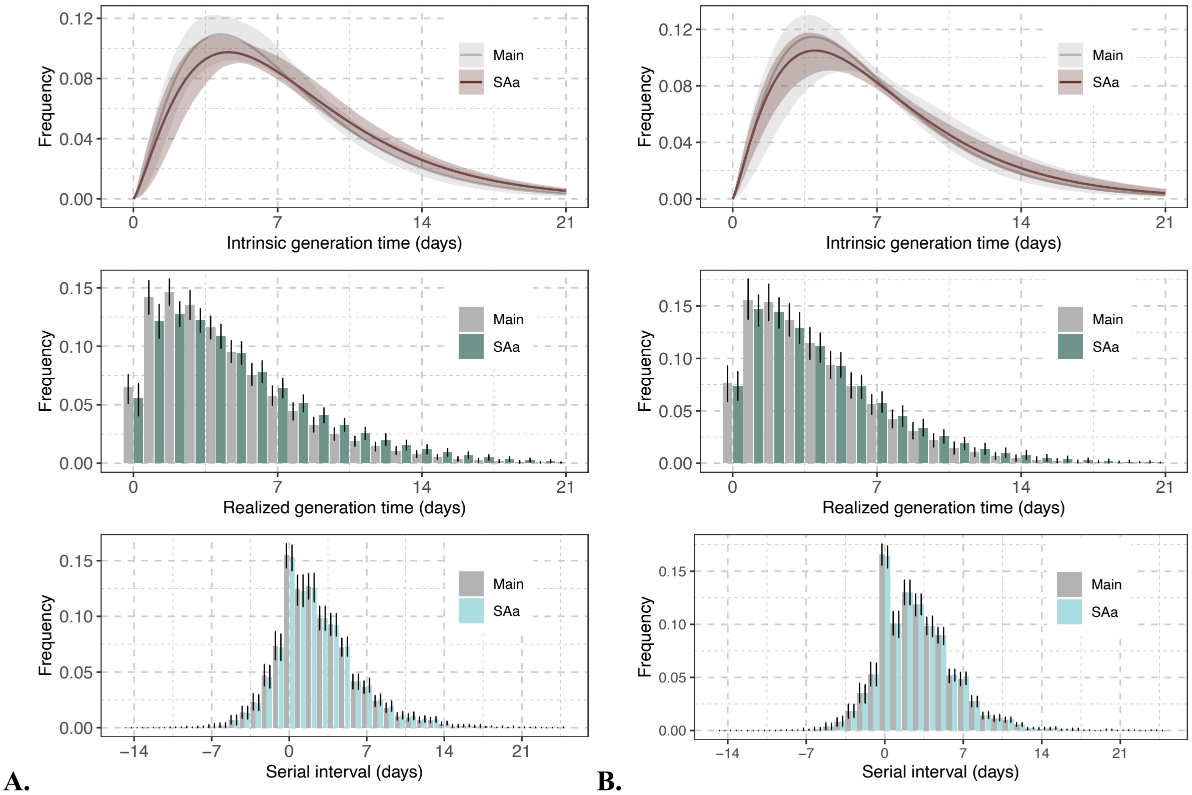

Figure S6. Comparison between baseline analysis and results obtained with sensitivity analysis a), using an alternative method for the imputation of infection dates for asymptomatic individuals. A. Alpha variant. **B.** Delta Variant.

Table S7 shows statistics on the posterior distributions of parameters for the intrinsic generation time:

Table S7. Statistics on the posterior distributions of parameters for the intrinsic generation time in sensitivity analysis a).

Alpha	Shape variance	0.06
	Scale variance	0.07
	Covariance	-0.048
Delta	Shape variance	0.03
	Scale variance	0.11
	Covariance	-0.036

b) Distribution of the incubation period – I

In this sensitivity analysis, we reassigned infectious dates according to the baseline method, but using a different probability density function of the incubation period P_S in Equation 1. We considered a gamma-distributed estimate for Ps with shape 2.08 and scale 3.03 as derived for ancestral lineages in [S2]. Table S8 and Figure S7 show that results obtained in this sensitivity analysis are in line with the baseline.

Table S8. Estimates for the intrinsic and realized generation time and serial intervals using an alternative distribution of incubation periods estimated for ancestral lineages in [S2].

		**ALPHA**	**DELTA**
**INTRINSIC GENERATION TIME**	mean (95%CrI) [days]	7.48 (6.72-8.48)	7.38 (6.31-8.86)
	shape mean (95%CrI)	2.46 (2.19-3.03)	2.39 (2.06-2.92)
	scale mean (95%CrI)	3.05 (2.58-3.5)	3.1 (2.58-3.64)
**REALIZED GENERATION TIME**	mean (95%CrI) [days]	4.76 (4.60-4.92)	4.66 (4.45-4.88)
**SERIAL INTERVAL**	mean (95%CrI) [days]	2.14 (1.98-2.32)	2.28 (2.12-2.43)

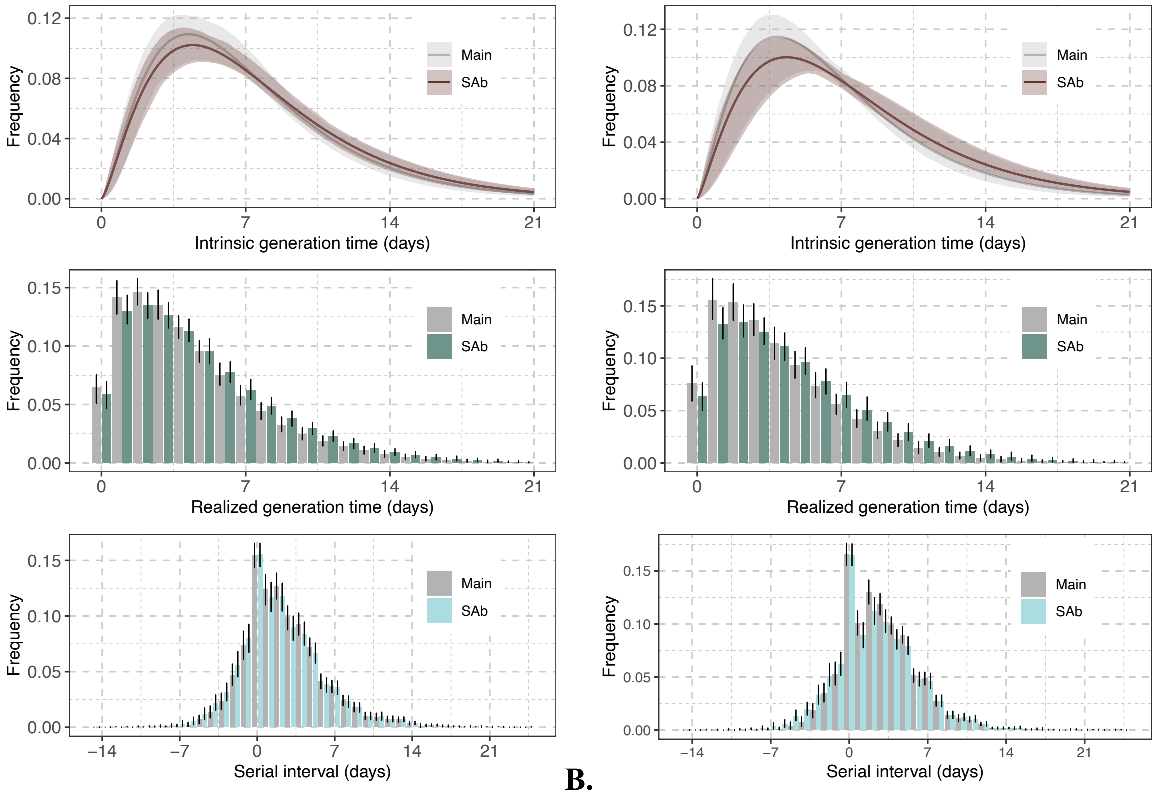

A. **B.**

Figure S7. Comparison between baseline analysis and results obtained with sensitivity analysis b), using an alternative distribution of incubation periods estimated for ancestral lineages in [S2]. A. Alpha variant. **B.** Delta Variant.

Table S9 shows statistics on the posterior distributions of parameters for the intrinsic generation time:

Table S9. Statistics on the posterior distributions of parameters for the intrinsic generation time in sensitivity analysis b).

Alpha	Shape variance	0.03
	Scale variance	0.06
	Covariance	-0.026
Delta	Shape variance	0.05
	Scale variance	0.08
	Covariance	-0.031

c) Distribution of the incubation period – II

Similarly to SA b), we considered a further alternative for the gamma-distributed estimate for Ps with shape 4.23 and scale 1.23, as derived for ancestral lineages in [S3]. Table S10 and Figure S8 show that results obtained in this sensitivity analysis are in line with the baseline.

Table S10. Estimates for the intrinsic and realized generation time and serial intervals using an alternative distribution of incubation periods estimated for ancestral lineages in [S3].

		**ALPHA**	**DELTA**
**INTRINSIC GENERATION TIME**	mean (95%CrI) [days]	7.1 (6.22-8.45)	6.8 (5.8-8.46)
	shape mean (95%CrI)	2.51 (2.25-3.07)	2.45 (2.11-2.94)

	scale mean (95%CrI)	2.84 (2.35-3.42)	2.79 (2.27-3.46)
**REALIZED GENERATION TIME**	mean (95%CrI) [days]	4.39 (4.25-4.56)	4.22 (4.05-4.40)
**SERIAL INTERVAL**	mean (95%CrI) [days]	2.48 (2.35-2.62)	2.61 (2.46-2.72)

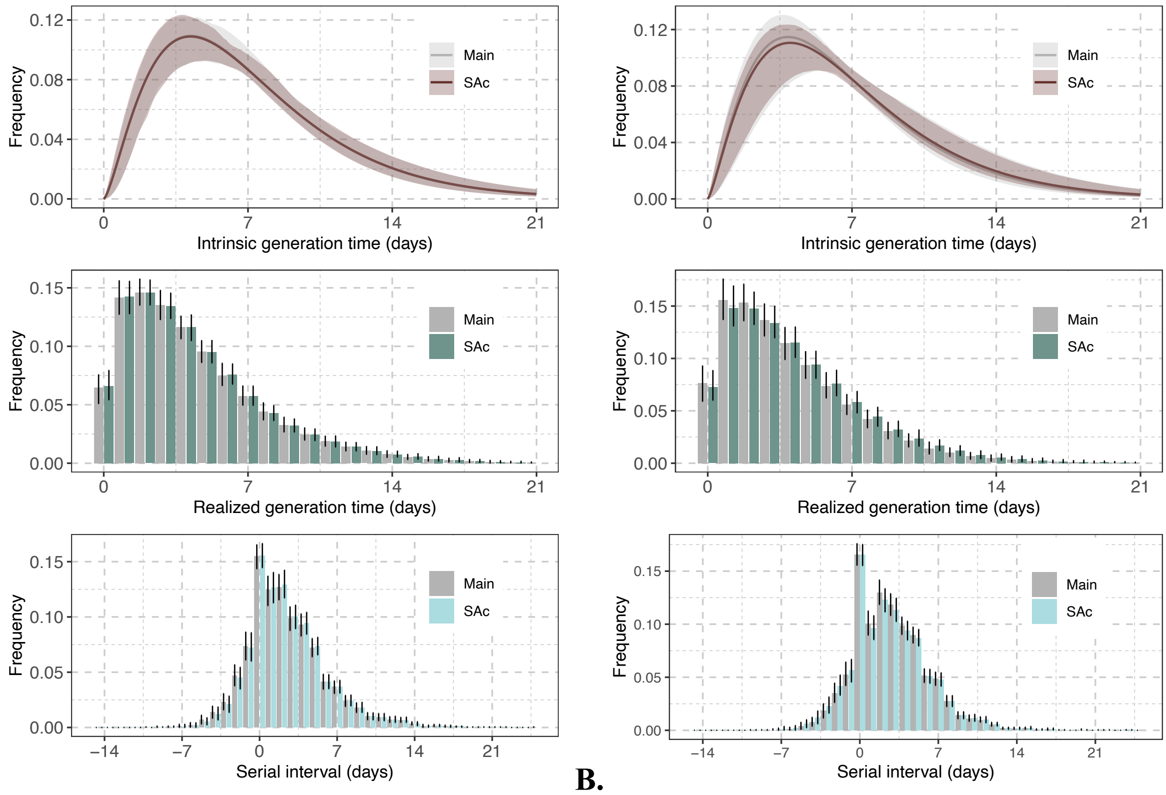

A. **B.**

Figure S8. Comparison between baseline analysis and results obtained with sensitivity analysis c), using an alternative distribution of incubation periods estimated for ancestral lineages in [S3]. A. Alpha variant. **B.** Delta Variant.

Table S11 shows statistics on the posterior distributions of parameters for the intrinsic generation time:

Table S11. Statistics on the posterior distributions of parameters for the intrinsic generation time in sensitivity analysis c).

Alpha	Shape variance	0.04
	Scale variance	0.08
	Covariance	-0.026
Delta	Shape variance	0.04
	Scale variance	0.10
	Covariance	-0.036

d) Reduced transmissibility for asymptomatic individuals

In this sensitivity analysis, we consider a halved transmissibility for asymptomatic individuals [S11] by modifying Equation 7 as follows:

$$\rho_i(t) = \begin{cases} \varphi_i & if\ t < t_{v,1} + 14 \\ \rho\varphi_i & if\ t \geq\ t_{v,1} + 14 \end{cases} \qquad \text{(Eq. 11)}$$

Where φ_i is 1 if I is symptomatic and 0.5 if asymptomatic. Table S12 and Figure S9 show that results obtained in this sensitivity analysis are in line with the baseline.

Table S12. Estimates for the intrinsic and realized generation time and serial intervals using a halved transmissibility for asymptomatic individuals.

		ALPHA	DELTA
INTRINSIC GENERATION TIME	mean (95%CrI) [days]	7.24 (6.6-8.52)	6.62 (5.81-8.25)
	shape mean (95%CrI)	2.56 (2.17-3.03)	2.43 (2.15-2.77)
	scale mean (95%CrI)	2.85 (2.39-3.43)	2.74 (2.29-3.36)
REALIZED GENERATION TIME	mean (95%CrI) [days]	4.51 (4.37-4.67)	4.06 (3.92-4.22)
SERIAL INTERVAL	mean (95%CrI) [days]	2.49 (2.37-2.62)	2.73 (2.62-2.85)

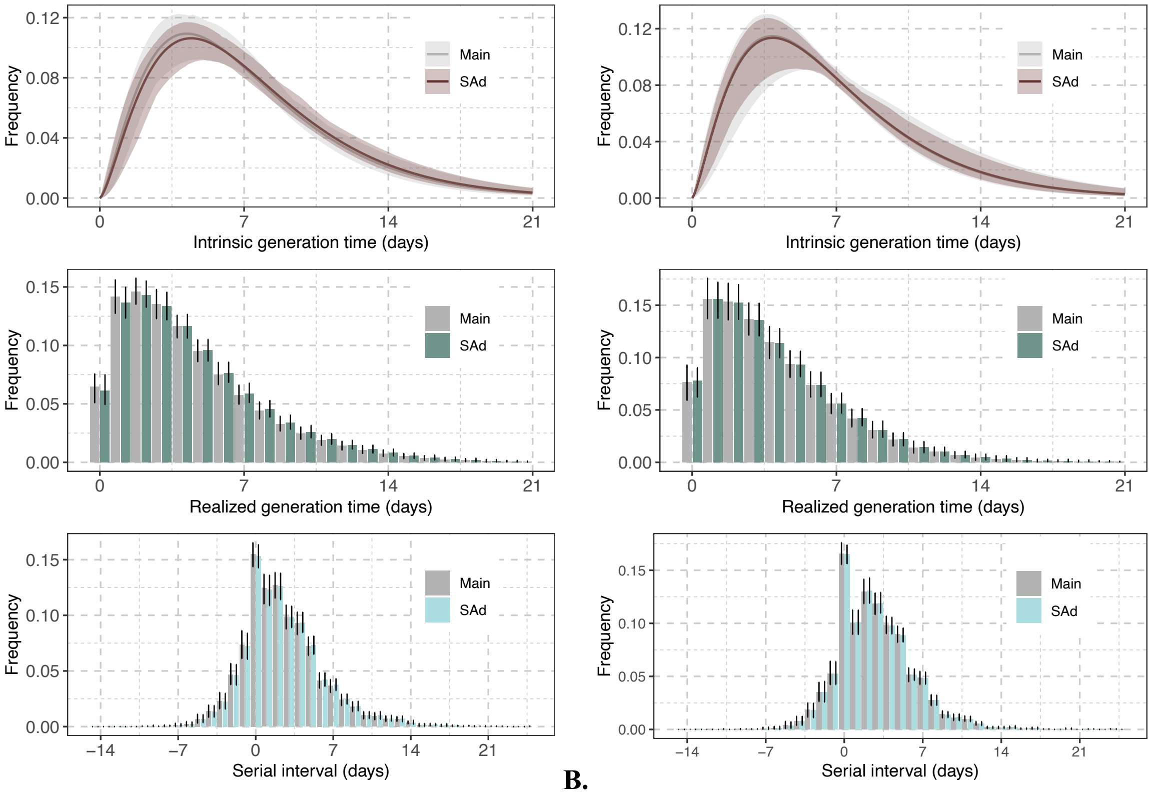

A. **B.**

Figure S9. Comparison between baseline analysis and results obtained with sensitivity analysis d), using a halved transmissibility for asymptomatic individuals. A. Alpha variant. **B.** Delta Variant.

Table S13 shows statistics on the posterior distributions of parameters for the intrinsic generation time:

Table S13. Statistics on the posterior distributions of parameters for the intrinsic generation time in sensitivity analysis d).

Alpha	Shape variance	0.04
	Scale variance	0.07
	Covariance	-0.041
Delta	Shape variance	0.03
	Scale variance	0.07
	Covariance	-0.021

e) Protection from previous infection in a fraction of undiagnosed household members

In this sensitivity analysis, we assume that a fraction of individuals who were undiagnosed were not susceptible to infection due to immunity conferred by previous SARS-CoV-2 infection. Using previous estimates of the cumulative SARS-CoV-2 attack rate in Italy before the Alpha and the Delta waves [S12], we assume that 15% of undiagnosed household cases during the Alpha period and 20% of undiagnosed household cases during the Delta period were immune. These cases were randomly sampled and removed from set of j for each of the Z repetitions of the MCMC procedure. The absence of these cases impacts on the component of Q_j of the likelihood in Equation 8. Table S14 and Figure S10 show that results obtained in this sensitivity analysis are in line with the baseline.

Table S14. Estimates for the intrinsic and realized generation time and serial intervals when assuming that 15% of undiagnosed cases in the Alpha period and 20% of undiagnosed cases in the Delta period were protected from infection via natural immunity from previous infection.

		**ALPHA**	**DELTA**
**INTRINSIC GENERATION TIME**	mean (95%CrI) [days]	7.23 (6.39-8.57)	6.52 (5.54-8.43)
	shape mean (95%CrI)	2.48 (2.26-2.87)	2.45 (2.13-2.87)
	scale mean (95%CrI)	2.92 (2.45-3.44)	2.75 (2.29-3.33)
**REALIZED GENERATION TIME**	mean (95%CrI) [days]	4.41 (4.27-4.56)	4.06 (3.89-4.25)
**SERIAL INTERVAL**	mean (95%CrI) [days]	2.43 (2.29-2.58)	2.75 (2.63-2.89)

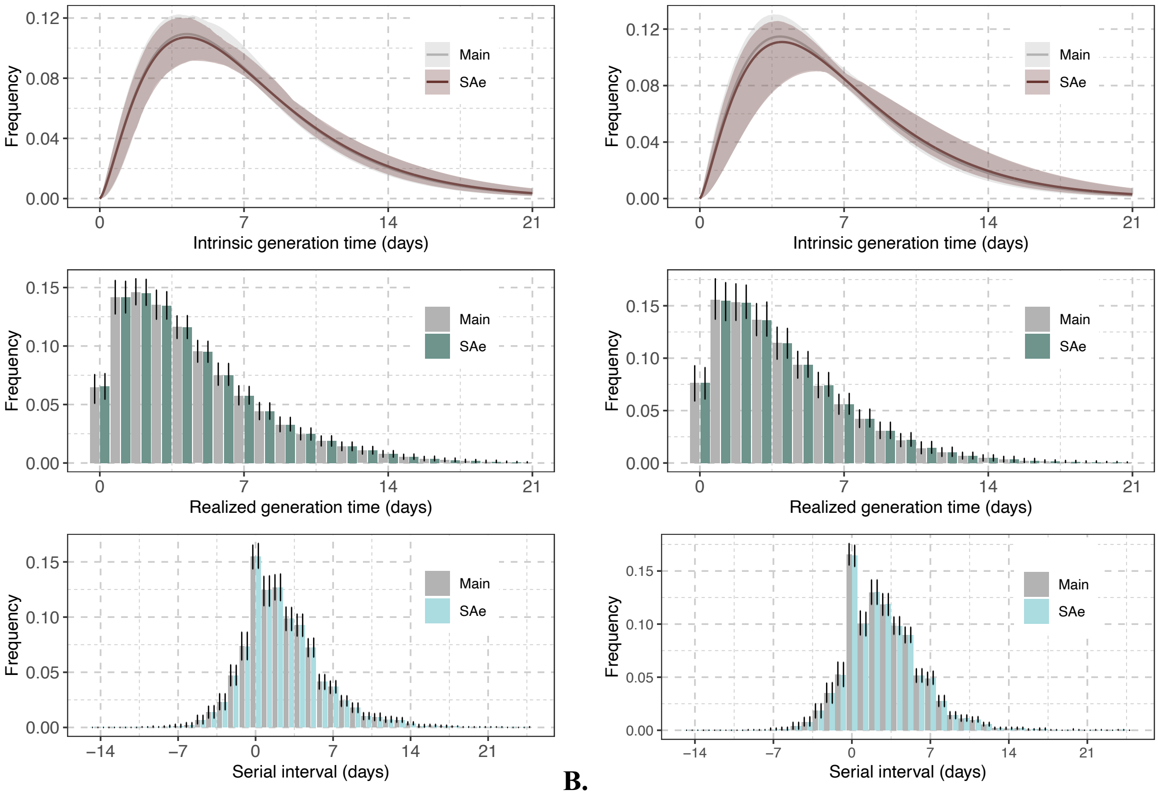

A. **B.**

Figure S10. Comparison between baseline analysis and results obtained with sensitivity analysis e), assuming that 15% of undiagnosed cases in the Alpha period and 20% of undiagnosed cases in the Delta period were protected from infection via natural immunity from previous infection. A. Alpha variant. **B.** Delta Variant.

Table S15 shows statistics on the posterior distributions of parameters for the intrinsic generation time:

Table S15. Statistics on the posterior distributions of parameters for the intrinsic generation time in sensitivity analysis e).

Alpha	Shape variance	0.03
	Scale variance	0.07
	Covariance	-0.024
Delta	Shape variance	0.04
	Scale variance	0.08
	Covariance	-0.022

f) No protection from infection outside the household during quarantine

In this sensitivity analysis, we assume that the imposed quarantine period after the first positive diagnosis would not impact the force of infection from outside the household (i.e., $q(t) = 1$ for any value of t in Equation 4b). Table S16and Figure S11 show that results obtained in this sensitivity analysis are in line with the baseline.

Table S16. Estimates for the intrinsic and realized generation time and serial intervals when assuming no protection from outside infection during the quarantine period.

		**ALPHA**	**DELTA**
**INTRINSIC GENERATION TIME**	mean (95%CrI) [days]	6.22 (5.77-6.65)	5.95 (5.28-6.69)

	shape mean (95%CrI)	2.48 (2.24-2.75)	2.41 (2.15-2.73)
	scale mean (95%CrI)	2.51 (2.18-2.89)	2.48 (2.06-2.95)
**REALIZED GENERATION TIME**	mean (95%CrI) [days]	4.09 (3.96-4.21)	3.84 (3.70-3.97)
**SERIAL INTERVAL**	mean (95%CrI) [days]	2.25 (2.1-2.38)	2.61 (2.5-2.72)

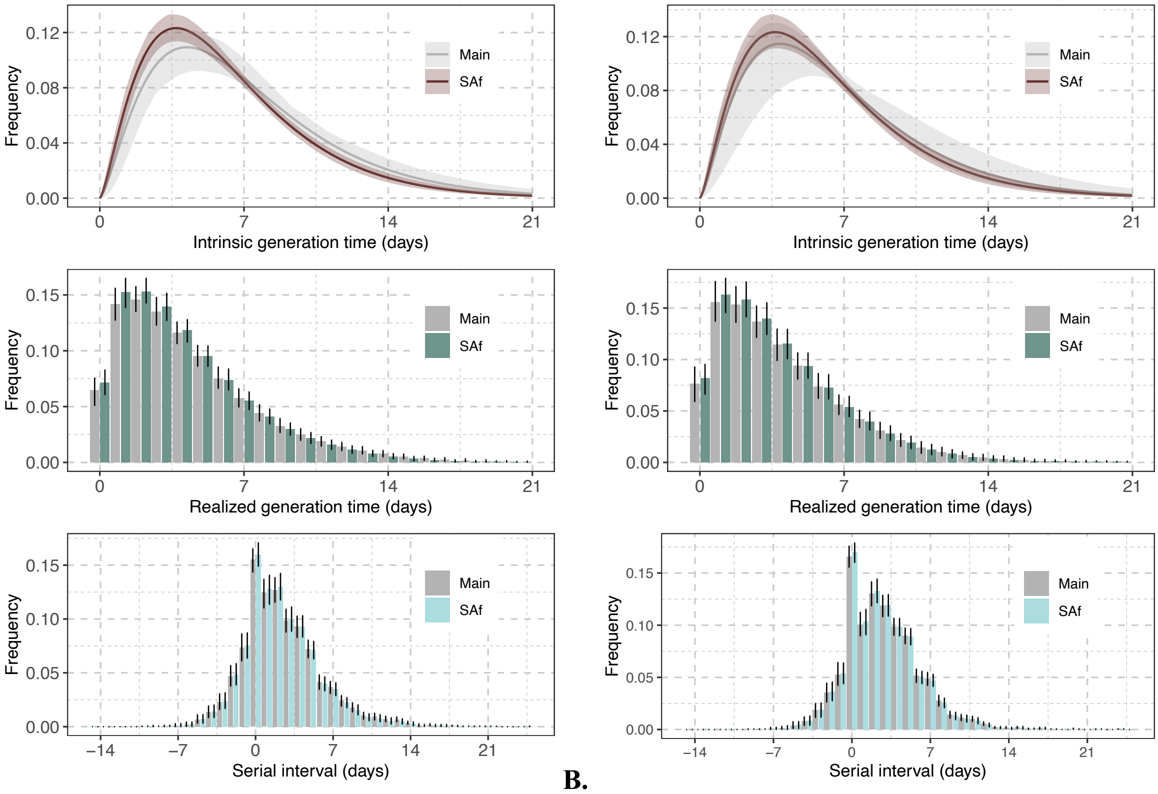

A. **B.**

Figure S11. Comparison between baseline analysis and results obtained with sensitivity analysis f), assuming no protection from outside infection during the quarantine period. A. Alpha variant. **B.** Delta Variant.

Table S17 shows statistics on the posterior distributions of parameters for the intrinsic generation time:

Table S17. Statistics on the posterior distributions of parameters for the intrinsic generation time in sensitivity analysis f).

Alpha	Shape variance	0.02
	Scale variance	0.03
	Covariance	-0.021
Delta	Shape variance	0.02
	Scale variance	0.05
	Covariance	-0.025

References

S1. J Hellewell, TW Russell, The SAFER Investigators and Field Study Team. et al. Estimating the effectiveness of routine asymptomatic PCR testing at different frequencies for the detection of SARS-CoV-2 infections. BMC Medicine. 2021; 19, 106. https://doi.org/10.1186/s12916-021-01982-x

S2. S Hu, W Wang, Y Wang, M Litvinova, K Luo, L Ren et al. Infectivity, susceptibility, and risk factors associated with SARS-CoV-2 transmission under intensive contact tracing in Hunan, China. Nature communications, 2021; 12(1): 1-11.

S3. Q Li, X Guan, P Wu, X Wang, L Zhou, Y Tong et al. Early transmission dynamics in Wuhan, China, of novel coronavirus–infected pneumonia. New England journal of medicine, 2020: 382:1199-1207

S4. G Guzzetta, C Minosse, R Pisapia, E Giombini, A Mammone, F Vairo, et al. Household transmission and disease transmissibility of a large HAV outbreak in Lazio, Italy, 2016–2017. Epidemics, 2019; 29, 100351.

S5. F Riccardo, M Ajelli, XD Andrianou, A Bella, M Del Manso, M Fabiani, et al.; COVID-19 working group. Epidemiological characteristics of COVID-19 cases and estimates of the reproductive numbers 1 month into the epidemic, Italy, 28 January to 31 March 2020. Euro Surveill. 2020. Dec;25(49):2000790. 10.2807/1560-7917.ES.2020.25.49.2000790

S6. F Riccardo, G Guzzetta, A. Mateo Urdiales, M. Del Manso, XD Andrianou, A Bella, et al. COVID-19 response: effectiveness of weekly rapid risk assessments, Italy. Bulletin of the World Health Organization, 2022; 100(2): 161.

S7. M Fabiani, M Puopolo, C Morciano, M Spuri, SS Alegiani, A Filia, F D'Ancona, et al. Effectiveness of mRNA vaccines and waning of protection against SARS-CoV-2 infection and severe covid-19 during predominant circulation of the delta variant in Italy: retrospective cohort study. BMJ 2022;376.

S8. F Menegale, M Manica, A Zardini, G Guzzetta, V Marziano, V d'Andrea, et al. Waning of SARS-CoV-2 vaccine-induced immunity: A systematic review and secondary data analysis. doi: https://doi.org/10.1101/2022.07.04.22277225

S9. RJ Harris, JA Hall, A Zaidi, NJ Andrews, JK Dunbar, G Dabrera. Effect of Vaccination on Household Transmission of SARS-CoV-2 in England. New England journal of medicine, 2021: 385(8): 759-760.

S10. M Lipsitch & R Kahn. Interpreting vaccine efficacy trial results for infection and transmission. Vaccine, 2021: 39(30): 4082-4088.

S11. D McEvoy, C McAloon, A Collins, et al Relative infectiousness of asymptomatic SARS-CoV-2 infected persons compared with symptomatic individuals: a rapid scoping review BMJ Open, 2021: 11:e042354.

S12. V Marziano, G Guzzetta, A Mammone et al. The effect of COVID-19 vaccination in Italy and perspectives for living with the virus. Nat Commun, 2021: 12: 7272
